# Sex differences in sleep deficits in mice with an autism-linked Shank3 mutation

**DOI:** 10.1186/s13293-024-00664-6

**Published:** 2024-10-28

**Authors:** Elizabeth Medina, Michael J. Rempe, Christine Muheim, Hannah Schoch, Kristan Singletary, Kaitlyn Ford, Lucia Peixoto

**Affiliations:** 1 Department of Translational Medicine and Physiology, Sleep and Performance Research Center, Elson S. Floyd College of Medicine, Washington State University, Spokane, WA USA; 2 Department of Integrative Physiology and Neuroscience, Washington State University, Pullman, WA USA

**Keywords:** Development, Sex differences, Autism spectrum disorder, Sleep, SHANK3

## Abstract

**Background:**

Insomnia is more prevalent in individuals with Autism Spectrum Disorder (ASD), can worsen core-symptoms and reduces quality of life of both individuals and caregivers. Although ASD is four times more prevalent in males than females, less is known about sex specific sleep differences in autistic individuals. Recent ASD studies suggest that sleep problems may be more severe in females, which aligns with the sex bias seen in insomnia for the general population. We have previously shown that male mice with a mutation in the high confidence ASD gene Shank3, Shank3^∆C^, recapitulate most aspects of the ASD insomnia phenotype. The objective of the present study was to leverage the Shank3^∆C^ model to investigate sex-specific effects in sleep using polysomnography.

**Methods:**

Adult male and female Shank3^∆C^ and wildtype (WT) littermates were first recorded for 24 h of baseline recordings. Subsequently, they were sleep deprived (SD) for five hours via gentle handling and allowed 19 h of recovery sleep to characterize the homeostatic response to SD. Vigilance states (rapid eye movement (REM) sleep, non-rapid eye movement (NREM) sleep and wake) were assigned by manual inspection using SleepSign. Data processing, statistical analysis and visualization were conducted using MATLAB.

**Results:**

Sex and genotype effects were found during baseline sleep and after SD. At baseline, male Shank3^∆C^ mice sleep less during the dark period (active phase) while female Shank3^∆C^ mice sleep less during the light period (rest phase) and sleep more during the dark period. Both male and female Shank3^∆C^ mice show reduced spectral power in NREM sleep. We detect a significant effect of sex and genotype in sleep onset latency and homeostatic sleep pressure (sleepiness). In addition, while male Shank3^∆C^ mice fail to increase sleep time following SD as seen in WT, female Shank3^∆C^ mice decrease sleep time.

**Conclusions:**

Overall, our study demonstrates sex differences in sleep architecture and homeostatic response to SD in adult Shank3^∆C^ mice. Thus, our study demonstrates an interaction between sex and genotype in Shank3^∆C^ mice and supports the use of the Shank3^∆C^ model to better understand mechanisms contributing to the sex differences in insomnia in ASD in clinical populations.

**Supplementary Information:**

The online version contains supplementary material available at 10.1186/s13293-024-00664-6.

## Plain language summary

Sleep problems are common in people with Autism Spectrum Disorder (ASD) and can make their condition worse, impacting both their lives and those of their caregivers. Historically ASD has been diagnosed more often in males and much less is known about the female phenotype. However, recent studies suggest that sleep problems may be more severe in autistic females. We previously shown that the ASD mouse model, Shank3^∆C^, display an autism like sleep phenotype. Using this model, we examine sex specific deficits in sleep. We investigated canonical measures of sleep during undisturbed conditions (sleep architecture) and after sleep deprivation (sleep homeostasis).

Our findings revealed that male Shank3^∆C^ mice slept less during their active period while female Shank3^∆C^ mice slept less during their rest period and more during their active period. In addition, both male and female Shank3^∆C^ mice displayed lower sleep quality. After sleep deprivation, both male and female Shank3^∆C^ mice took longer to fall asleep despite accumulation of sleep pressure, however, differences in sleep time after sleep deprivation differed based on sex. In healthy controls, sleep time increases after sleep deprivation. Male Shank3^∆C^ mice showed no such increase in sleep time, while female Shank3^∆C^ mice slept even less. Our results suggest sex specific differences in two hallmark measures of sleep, architecture and sleep homeostasis in this ASD model. These results highlight the importance of awareness of sex specific differences when studying sleep in ASD as well as eventual treatment or interventions.

**Supplementary Information:**

The online version contains supplementary material available at 10.1186/s13293-024-00664-6.

## Highlights

- At baseline, Shank3^∆C^ mice have a time-of-day dependent reduction in sleep time. Male Shank3^∆C^ mice had reduced sleep during the active phase, while female mice had reduced sleep during the rest phase but increased sleep during the active phase.
- Shank3^∆C^ mice have a reduction of EEG spectral power during non-rapid eye movement sleep, suggesting reduced sleep synchrony or quality.
- Both male and female Shank3^∆C^ mice show an impaired response to sleep deprivation. While male Shank3^∆C^ mice fail to increase sleep time after sleep deprivation, female Shank3^∆C^ mice decrease sleep time after sleep deprivation.
- After sleep deprivation, both male and female Shank3^∆C^ mutant mice have a delayed sleep onset despite accumulation of sleep pressure similar to wildtype animals.

**Supplementary Information:**

The online version contains supplementary material available at 10.1186/s13293-024-00664-6.

## Background

Sleep problems are more prevalent among individuals with Autism Spectrum Disorder (ASD) compared to those with typical development, affecting up to 93% of individuals [30]. These problems include difficulties falling asleep, sleep fragmentation, and a reduction in sleep time, which are defining symptoms of insomnia. Insomnia during development has been correlated with long term behavioral deficits and have been shown to worsen core symptoms of ASD [9, 15, 16, 37]. However, less is known about the relationship between sex and sleep problems in ASD. In typical development, insomnia is more prevalent in females [18, 26, 27, 41], thus, it is not surprising that recent studies show that autistic females may have more sleep-related disturbances than males [4, 10, 12]. Understanding how sleep problems present differently in autistic males and females will allow for better targeted interventions to treat insomnia in ASD.

Mouse models allow us to better determine the nature of sleep deficits and the evaluate the homeostatic response to sleep deprivation (SD) using objective measures. Sleep is a homeostatic process. The need to sleep (sleepiness) increases as a function of time spent awake, driving subsequent compensatory recovery sleep, which in turn discharges sleep pressure. Since abnormalities on the homeostatic response to SD are an essential aspect of insomnia, evaluating it in preclinical studies is important for the translational potential of the findings. Genetic syndromes associated with high rates of ASD have been recapitulated in animal models and have provided valuable insight into the molecular underpinnings of the disorder [24, 39]. We have previously shown that male mice carrying a mutation in high-confidence ASD risk gene Shank3 (Shank3^∆C^) display sleep abnormalities starting as young as postnatal day 24 and continue into adulthood [17, 25]. Male Shank3^∆C^ mice sleep less and have a reduction of spectral power during non-rapid eye movement (NREM) sleep. In addition, they take a long time to fall asleep despite being sleepy and do not show the expected sleep rebound (increase in sleep time) after sleep loss. Recent studies using Shank3 mutant mice suggest both males and females display sleep deficits [5, 21]. However, previous studies have not compared males and females directly, nor evaluated the homeostatic response to SD. To better understand sleep deficits associated with insomnia, it is essential to consider both sexes independently and to investigate the homeostatic aspect of sleep regulation [31, 36].

The objective of this study is to investigate sex differences in sleep architecture and sleep homeostasis in Shank3^∆C^ mice using electroencephalography (EEG) and electromyography (EMG). We find that Shank3^∆C^ mice display sleep deficits during undisturbed baseline sleep and in the response to SD in a sex dependent manner. At baseline, we find that female Shank3^∆C^ mice sleep less during the light period but more during the dark period, while male Shank3^∆C^ mice only sleep less during the dark period. In response to SD, Shank3^∆C^ mice have sex specific differences in the homeostatic response to SD. Male Shank3^∆C^ mice fail to increase sleep amounts in response to SD, while female Shank3^∆C^ mice have a decrease in time in NREM sleep, opposite the typical homeostatic response to SD. In addition, both male and female Shank3^∆C^ mice take longer to fall asleep despite being sleepy. Overall, our study demonstrates that sex and genotype interact to modify sleep architecture and the homeostatic response to SD in adult Shank3^∆C^ mice.

## Methods

### Animals

Heterozygous Shank3^∆C^ mice were obtained from Dr. Paul Worley at Johns Hopkins University. Shank3^∆C^ breeding pairs were established to obtain wildtype (WT) and Shank3^∆C^ littermates. Mice were housed at 24 ± 1 °C on a 12:12 h light: dark cycle with food and water ad libitum. All experimental procedures were approved by the Institutional Animal Care and Use Committee of Washington State University and conducted in accordance with National Research Council guidelines and regulations for experiments in live animals.

### Surgical procedures

A total of 28 animals were equipped with EEG/EMG for this study. Adult (8–12 weeks old) male and female mice (n = 7 per sex and genotype) were anesthetized and implanted with four electroencephalographic and two electromyographic electrodes as previously described [25]. Briefly, four stainless steel screws (BC‐002MP188, Bellcan International Corp, Hialeah, FL) were placed bilaterally over frontal (two) and parietal (two) cortices, and EMG electrodes were inserted bilaterally into the nuchal muscles. EEG electrode placement was secured with dental composite, the mice were individually housed and allowed a minimum of five days of recovery from surgery prior to habituation to the recording environment. Animals received dexamethasone (1 mg/kg), enrofloxacin (5 mg/kg) and carprofen (5 mg/kg) prior to surgery and as postop treatments for an additional two days. Buprenorphine (0.1/mg/kg) was given as analgesic.

### EEG/EMG data acquisition and processing

Animals were connected to a lightweight, flexible tether and allowed five days to habituate to the tether and recording environment. Every animal was recorded for a single 48-h recording period. Recordings started at lights on (ZT 0, day 1) followed by five hours of SD at the beginning of lights on (ZT 0, day 2). SD was followed by 19 h of undisturbed recovery sleep. SD was conducted manually via gentle handing, when necessary, animals were gently stroked with a soft brush to ensure animals remained awake [28]. SD efficiency was calculated as the percentage of wake epochs for the total duration of SD. SD efficiencies and standard error of the mean per group were as follows: Male WT: 98% ± 1%, Male Shank3^∆C^: 96% ± 1%, Female WT: 98% ± 1%, Female Shank3^∆C^: 97% ± 1%, confirming our animals were awake for the majority of the time.

EEG and EMG data were collected using custom built electrophysiological amplifiers (Intan Technologies, RHD amplifier chips) via a lightweight counterbalanced cable and downsampled to 250 Hz. EEG and EMG data were high and low band pass filtered at 0.05 and 50 Hz and 15 and 30 Hz, respectively, with a Notch filter set to 60 Hz. Data was manually scored via visual inspection in four second epochs by an experimenter blinded to conditions using SleepSign (Kissei Comtec Co., LTD, Nagano, Japan). Every epoch was scored as one of three states: wake, non-rapid eye movement (NREM) sleep, or rapid eye movement (REM) sleep. For bout duration and bout number analyses, only bouts of at least two epochs (eight seconds) were included. Latency to NREM sleep after SD was defined as time elapsed from release to recovery sleep to the first bout of NREM sleep (bout ≥ 28 s). Raw EEG data is available at https://sleepdata.org/datasets/shank3zz.

Fast Fourier transformation data was exported from SleepSign for each animal and used in downstream analysis. Data analysis, visualization, and statistics were performed using MATLAB (MathWorks). MATLAB code is available through Github (https://github.com/PeixotoLab/Shank3Z_sexdifferences). EEG epochs containing artifacts were excluded from spectral analysis. One male mutant animal was excluded following sleep deprivation due to extensive periods of artifacts and signal loss. The relative power spectra were defined as a percent of total power across vigilance states (0.05–50 Hz) for baseline and recovery sleep separately. EEG spectral power across either baseline or recovery sleep is displayed as mean plus 95% confidence intervals. Differences in spectral power were defined as lack of overlap between 95% confidence intervals as in our previously published work [17, 25]. Sleep pressure was defined as EEG spectral power in the delta range (0.5–4 Hz) of NREM sleep during the recovery period normalized to mean NREM sleep delta power of the last four hours of the light period at baseline.

### Statistical analysis

Repeated measures ANOVA using time as the repeated measure and genotype (WT vs. Shank3^∆C^ mice) and sex (male or female) as the between subjects’ variable was used for most measures (i.e., total sleep time, sleep latency, time in state, bout number, bout duration, homeostatic NREM delta power). Post hoc tests were performed if a main effect of sex or genotype or the interaction effects sex*time or genotype*time was present. Most post-hoc comparisons were unpaired t-tests except for post hoc comparisons for sleep pressure. Post hoc tests for sleep pressure (Fig. 3A) were repeated measures ANOVA to preserve the effect of time on sleep pressure discharge. Benjamini–Hochberg was used to correct for multiple comparisons in all post hoc tests. Analysis of time in state and associated measures (e.g., bout number and duration) was performed independently for the light period (ZT 0–12 or 6–12) and dark periods (ZT 13–24) because of the known differential effect of light on sleep parameters [11]. Testing for differences between baseline and sleep following SD was performed within sex and within genotype only using repeated measures ANOVA.

## Results

### Shank3^∆C^ mice display sex specific deficits in sleep timing at baseline

The goal of this study was to investigate whether the ASD mouse model, Shank3^∆C^, display sex specific deficits in sleep or the homeostatic response to acute SD. To do so, we performed EEG/EMG recordings under baseline conditions and following SD in male and female adult Shank3^∆C^ mice and their WT littermates (see Methods). We first investigated whether there were differences in sleep quantity during both the light period (when mice sleep the most, rest phase) and dark period (when mice sleep the least, active phase). Differences in sex and genotype were present during both the light and dark period. During the light period genotype differences are only present in Shank3^∆C^ female mice.

**Fig. 1:**
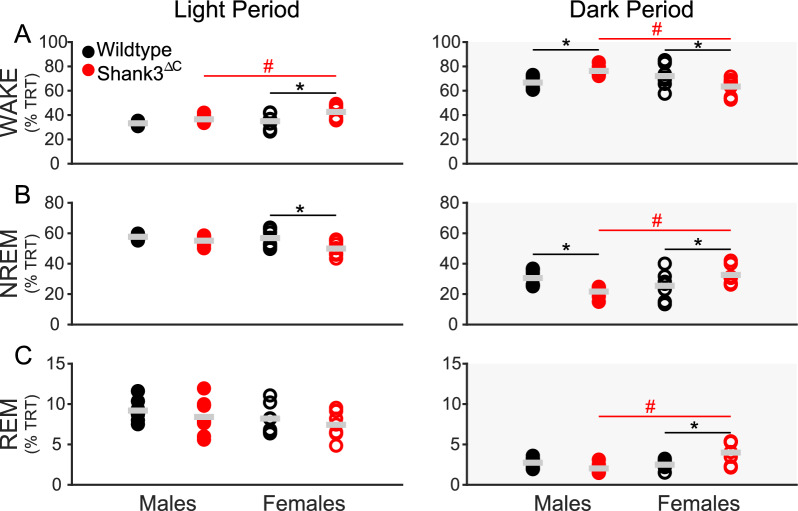
Sleep deficits in Shank3^∆C^ mice are dependent on time of day. Baseline time in state as percentage of total recording time (TRT). Wake, NREM sleep, and REM sleep during baseline light (white) and dark (gray) periods. N = 7/ sex/ genotype, mean value in gray bar. WT represented in black, Shank3^ΔC^ mice represented in red, filled represents males, unfilled represents females. # p-values < 0.05 across sex. *p-values < 0.05 across genotype for post hoc t-test with Benjamini–Hochberg correction A light period: Shank3^ΔC^ across sex p = 0.040 (Males: 36.5 ± 1.2%; Females: 42.6 ± 2.2%), females across genotype p = 0.010 (WT: 35.0 ± 2.3%; Mutant: 42.6 ± 2.2%).** A** dark period: males across genotype p = 0.020 (WT: 66.7 ± 2.0%; Mutant: 76.3 ± 1.5%), females across genotype p = 0.028 (WT: 72.1 ± 3.6%; Mutant: 63.4 ± 2.7%), Shank3^ΔC^ across sex p = 0.002 (Males: 76.3 ± 1.5%; Females: 63.4 ± 2.7%). B light period: females across genotype p = 0.009 (WT: 56.8 ± 2.1%; Mutant: 50.0 ± 1.8%). **B** dark period: males across genotype p = 0.018 (WT: 30.6 ± 1.9%; Mutant: 21.7 ± 1.4%), females across genotype p = 0.047 (WT: 25.4 ± 3.5%; Mutant: 32.6 ± 2.3%), Shank3^ΔC^ across sex p = 0.003 (Males: 21.7 ± 1.4%; Females: 32.6 ± 2.3%). **C** dark period: Shank3^ΔC^ across sex p = 0.001 (Males: 2.0 ± 0.2%; Females: 4.0 ± 0.5%), females across genotype p = 0.007 (WT: 2.5 ± 0.2%; Mutant: 4.0 ± 0.5%). Values in parentheses are mean ± SEM

Shank3^∆C^ female mice spend more time awake compared to WT females (Fig. 1A, left panel) and Shank3^∆C^ male mice and less time in NREM sleep compared to WT females (Fig. 1B, left panel). During the dark period, both male and female Shank3^∆C^ mice show differences, albeit in opposing directions. Shank3^∆C^ male mice spend more time awake than WT males (Fig. 1A, right panel) and less time in NREM sleep (Fig. 1B, right panel). Shank3^∆C^ female mice spend less time awake during the dark period compared to WT females (Fig. 1A) and more time in NREM sleep (Fig. 1B) and REM sleep (Fig. 1C). A sex difference in the Shank3^∆C^ mice is present in all three states during the dark period (Wake Fig. 1A, NREM sleep Fig. 1B, REM sleep Fig. 1C). 24-h time in state data can be found in Supplementary Figure S1. Full statistics for baseline measurements can be found in Additional file 2.

### Both Shank3^ΔC^ males and females display reduced NREM sleep quality

Next, we investigated differences in sleep quality, as they can have functional consequences even if sleep quantity only shows minor changes. Important aspects of sleep quality include alterations in sleep architecture and spectral power of vigilance states [20, 29]. Thus, we first investigated sleep architecture. We examined the number and duration of bouts per vigilance state to understand whether the reduction in time in state is driven by an inability to maintain a state (e.g., shorter bouts), the inability to enter a state (e.g., lower number of bouts) or a combination of both. We detect differences in bout number and bout duration across both light period and dark period. However, the genotype differences in both male and female Shank3^∆C^ in NREM sleep as seen in Fig. 1 are not supported by difference in bout number or duration (Supplementary Figure S2). All statistics for baseline measurements of time in state can be found in Additional file 2.

**Fig. 2:**
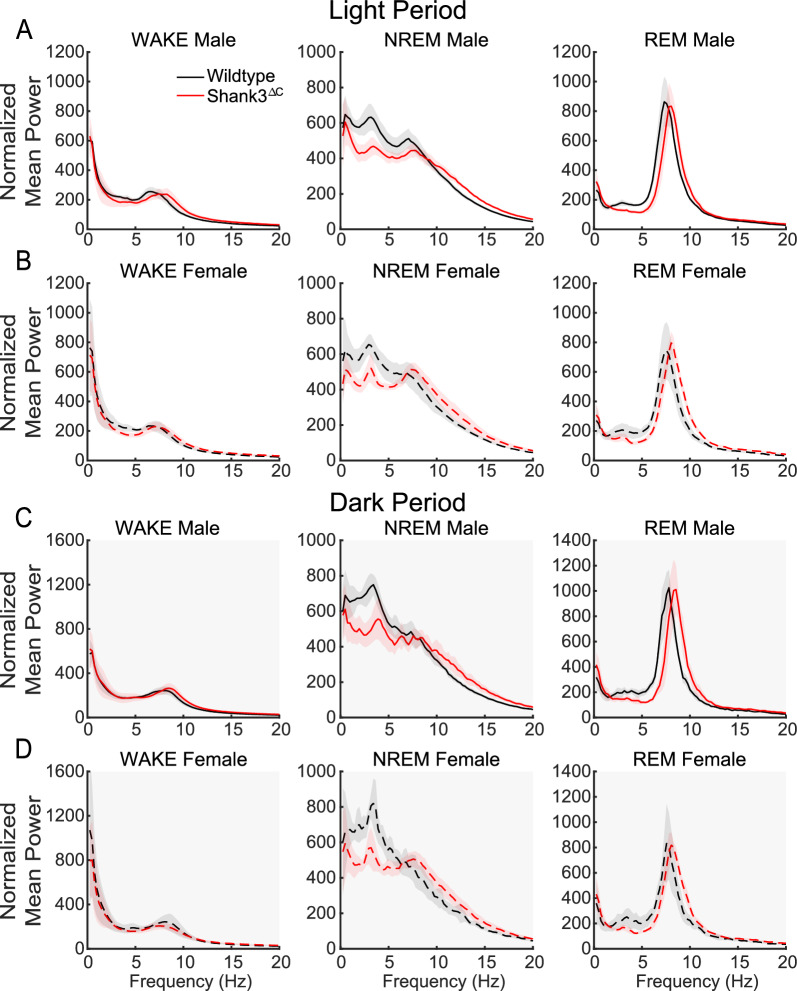
Shank3^∆C^ mice display altered spectral power at baseline. Spectral power normalized to total spectral power across 24 h of baseline. Light period** A**,** B**. Dark period** C**,** D**. 95% confidence intervals shaded in red and black. WT represented in black, Shank3^ΔC^ mice represented in red. Top row males (solid lines), bottom row females (dotted lines). N = 7/ sex/ genotype

Next, we investigated differences in sleep and wake state EEG spectral power. EEG spectral analysis is a well-established method to determine the strength or synchrony of a given state [1]. We have previously shown that male Shank3^∆C^ mice have a reduction in power in the delta range (0.5–4 Hz) of NREM sleep [17, 25]. Our data shows that under baseline conditions, both male and female Shank3^∆C^ mice have a reduction in NREM sleep delta EEG power compared to WT animals in both the light and dark period (Fig. 2). Thus, the reduction of NREM sleep EEG power intensity in Shank3 mutants is independent of sex.

### Shank3^ΔC^ male and female mice lack a homeostatic response to SD

**Fig. 3:**
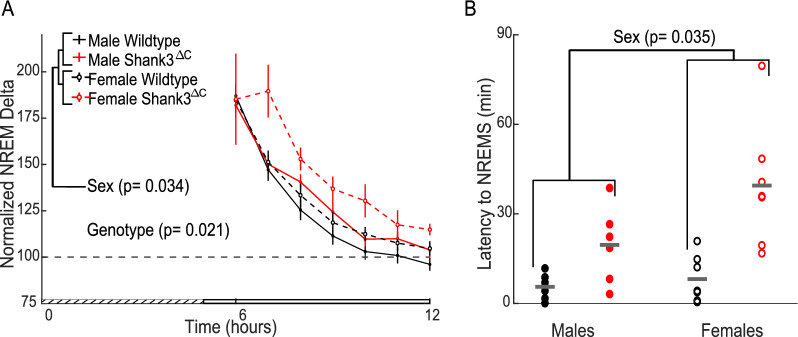
Shank3^ΔC^ mice have an increase in latency to sleep despite similar accumulation of sleep pressure following sleep deprivation. **A** Hourly NREM sleep delta power (0.5–4 Hz) after SD. Main effect of sex and genotype shown from initial repeated measures ANOVA. Error bars represent SEM, grated area represents the five hours of SD. **B** NREM sleep onset latency following SD. Mean value represented in gray bar. Repeated measures ANOVA main effect of sex shown in figure, main effect of genotype p = 1.614E -04, post hoc unpaired t-test with Benjamini–Hochberg correction: males across genotype p = 0.037 (WT: 5.6 ± 1.5 min; Mutant: 19.6 ± 5.2 min), females across genotype p = 0.012 (WT: 8.2 ± 3.0 min; Mutant: 39.5 ± 7.9 min). WT represented in black, Shank3^ΔC^ mutants represented in red (n = 7 WT males, 7 WT females, 6 Shank3^ΔC^ males, 7 Shank3^ΔC^ females). Values in parentheses are mean ± SEM

The homeostatic response to SD consists of a compensatory increase in sleep pressure and duration [3, 7]. NREM sleep delta EEG power after SD (normalized relative to baseline) is widely accepted as a reliable marker for sleep homeostasis of sleep pressure [2, 7, 13]. To investigate the homeostatic response to SD in Shank3^ΔC^ mice, we measured normalized delta EEG power in NREM sleep after SD. We detected a main effect for sex and genotype (Fig. 3A). Post hoc testing within sex across genotypes show a genotype effect for females (WT vs Shank3^ΔC^ p = 0.039) and a genotype*time effect for males (WT vs. Shank3^ΔC^ p = 0.014). However, neither of these p-values remained significant after multiple testing corrections. In addition, we measured sleep onset latency, as problems falling asleep despite being sleepy is a common symptom in ASD patients and in insomnia. Shank3^ΔC^ mice display an increase in sleep onset latency to NREM sleep following SD with a main effect of both sex and genotype (Fig. 3B). Post hoc t-tests within sex across genotype show a genotype effect for increased sleep latencies in both the females and males supporting that the genotype effect is stronger than the sex effect. Full statistics for Fig. 3 can be found in Additional file 2.

**Fig. 4:**
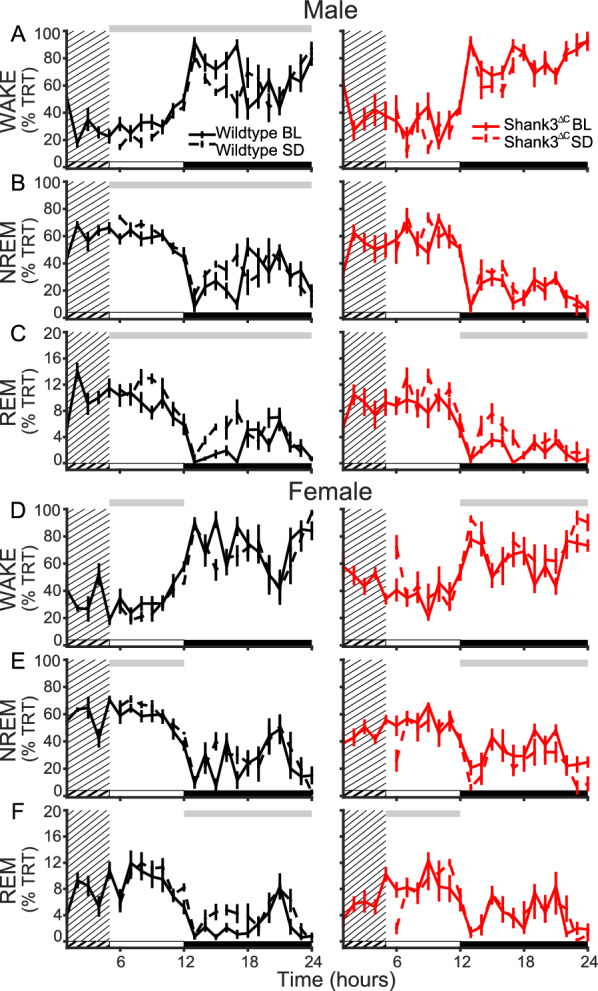
Shank3^ΔC^ mice lack a homeostatic response to SD. Total recording time (TRT) in wake (top row), NREM sleep (middle row) and REM sleep (bottom row) in recovery sleep compared to baseline. **A**–**C** Males. **D**–**F** Females. Gray bar above plots represents significance from repeated measures ANOVA across hour 6–12 (light period) or 13–24 (dark period). Light period represented by white bar, dark period black bar. Dashed line represents recovery sleep day, solid line represents baseline day, grated area represents the five hours of SD. WT represented in black, Shank3^ΔC^ mice represented in red (n = 7 WT males, 7 WT females, 6 Shank3^ΔC^ males, 7 Shank3^ΔC^ females). **A** WT light period for treatment F (6, 12) = 31.678, p = 1.110E-04, WT dark period for treatment *time F (11, 12) = 3.446, p = 3.078E-04. **B** WT NREM sleep light period for treatment F (6, 12) = 21.965, p = 001, WT NREM sleep dark period for treatment*time F (11, 12) = 3.313, p = 4.800E-04. **C** WT light period for treatment F (6, 12) = 8.007, p = 0.015, WT dark period for treatment F (11, 12) = 10.294, p = 0.008. Shank3^ΔC^ dark period for treatment F (11, 11) = 9.332, p = 0.011. **D** WT light period for treatment F (6, 11) = 4.896, p = 0.047. Shank3^ΔC^ dark period for treatment F (11, 12) = 6.128, p = 0.029**. E** WT light period for treatment F (6, 11) = 4.964, p = 0.046. Shank3^ΔC^ dark period for treatment F (11, 12) = 8.293, p = 0.014. **F** WT dark period for treatment F (6, 11) = 15.901, p = 0.002, Shank3^ΔC^ light period for treatment*time F (11, 12) = 2.332, p = 0.041

We then analyzed time spent in different vigilance states during recovery sleep period relative to baseline sleep. SD induces a sleep rebound or an increase in sleep time, typically in the dark period [3, 13]. WT males show a normal sleep rebound with a reduction in time in wake during the recovery period (Fig. 4A, left panel) accompanied by an increase in time in NREM sleep (Fig. 4B, left panel) and REM sleep (Fig. 4C, left panel). Male Shank3^ΔC^ mice do not show a decrease in wake, or an increase in NREM sleep, but do have an increase in REM sleep during the dark period (Fig. 4C, right panel), indicating an absence of NREM sleep rebound. WT females had an increase in wake during the light period (Fig. 4D, left panel), along with a decrease in NREM sleep (Fig. 4E, left panel) and a decrease in REM sleep (Fig. 4F, left panel), showing sleep rebound but selective to time of day. Female Shank3^ΔC^ mice had an increase in time in wake (Fig. 4D, right panel) along with a decrease in NREM sleep (Fig. 4E, right panel) and REM sleep (Fig. 4F, right panel). These results show that there is an absence of NREM sleep rebound (increase) in Shank3^ΔC^ males with a paradoxical decrease in NREM sleep after SD in the female Shank3^ΔC^ mice. Full statistics for Fig. 4 can be found in Additional file 2.

**Fig. 5:**
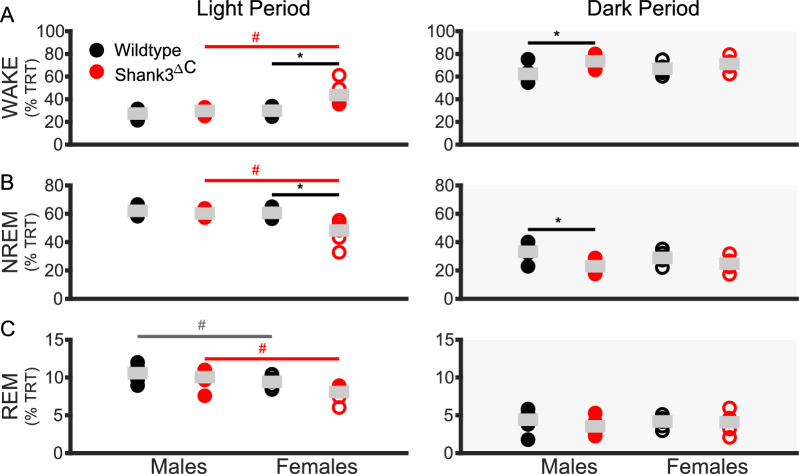
Shank3^ΔC^ mice have an altered response to SD. Recovery time in state as percentage of total recording time (TRT) in wake, NREM sleep, and REM sleep. Light period white background, dark period gray background. WT represented in black, Shank3^ΔC^ mice represented in red, filled represents males, unfilled represents females (n = 7 WT males, 7 WT females, 6 Shank3^ΔC^ males, 7 Shank3^ΔC^ females). Mean value in gray bar. # p-values < 0.05 across sex. * p-values < 0.05 across genotype for post hoc t-test with Benjamini–Hochberg correction **A** light period: Shank3^ΔC^ across sex p = 2.011E-04 (Males: 29.7 ± 1.0%; Females: 43.7 ± 2.1%), females across genotype p = 0.1.474E-04 (WT: 29.9 ± 0.8%; Mutant: 43.7 ± 2.1%), dark period: males across genotype p = 0.002 (WT: 62.4 ± 2.5%; Mutant: 73.5 ± 2.5%).** B** light period: Shank3^ΔC^ across sex p = 2.610E-04 (Males: 60.3 ± 1.0%; Females: 48.2 ± 3.0%), females across genotype p = 1.053E-04 (WT: 60.7 ± 1.1%; Mutant: 48.2 ± 3.0%), dark period: males across genotype p = 0.001 (WT: 33.2 ± 2.0%; Mutant: 23.0 ± 2.1%).** C** light period: WT across sex p = 0.049 (Males: 10.6 ± 0.4%; Females: 9.4 ± 0.3%), Shank3^ΔC^ across sex p = 0.005 (Males: 10.1 ± 0.5%; Females: 8.1 ± 0.4%). Values in parentheses are mean ± SEM

Since our previous analysis suggests that Shank3^∆C^ mice have problems with sleep rebound after SD, we directly compared time in state after SD across sex and genotype. During the remaining light period, compared to WT females and male Shank3^∆C^ mice, female Shank3^∆C^ mice spent more time awake and less time in NREM sleep (Fig. 5A, B). Unlike at baseline, there is a sex dependent reduction in REM sleep for both WT and Shank3^∆C^ mice (Fig. 5C).

During the dark period, male Shank3^∆C^ mice spent more time awake and less time in NREM sleep (Fig. 5A, B) compared to WT males. SD abolishes the differences in the dark period seen at baseline for female Shank3^∆C^ mice. 24-h time in state plots for recovery day can be found in Supplementary Figure S3. As seen in baseline these differences in time in state cannot be directly explained by changes in bout number and duration during the recovery period (Supplementary Figure S4). We also investigated the full spectral analysis of all vigilant states during the recovery period. The reduction in spectral power during NREM sleep seen at baseline is also observed during the recovery period after SD (Supplementary Figure S5). Full statistics for time in state on recovery day can be found in Additional file 2. Taken together with the spectral and sleep latency data from Fig. 3, and time in state data from Fig. 4, these data support that Shank3^ΔC^ mice display an altered homeostatic response to SD.

## Discussion

The present study uses the Shank3^∆C^ model of ASD to investigate the interaction between sex and genotype in baseline sleep and the homeostatic response to sleep loss using polysomnography. Our results demonstrate that male and female Shank3^∆C^ mice display sleep deficits at baseline in a sex dependent manner. Female Shank3^∆C^ mice sleep less during the light period (rest phase), and more during the dark period (active phase), while male Shank3^∆C^ mice sleep less only in the dark period (Fig. 1). In addition, both male and female Shank3^∆C^ mice have a reduction of EEG spectral power during NREM sleep (Fig. 2), which may be indicative of reduced sleep synchrony or quality [20, 23, 29, 38]. Our study also finds effects of genotype and sex on the homeostatic response to SD. Specifically, both male and female Shank3^∆C^ mice have an increase in sleep latency following SD, despite accumulation and discharge of sleep pressure (Fig. 3). In other words, they take longer to fall asleep despite being sleepy. In addition, female, but not male, Shank3^∆C^ mice have an increase in time in wake and decreased time in NREM sleep after SD, opposite the typical homeostatic response to SD which is characterized by an increase in sleep time (Fig. 4D, E). When we analyze time in state following SD, we find differences dependent on time of day. During the light period, we find sex and genotype differences in the Shank3^∆C^ mice. However, only genotype differences in the male Shank3^∆C^ mice are present in the dark period after SD (Fig. 5). Notably, the decrease in sleep in Shank3^∆C^ females during the light period become more pronounced after SD while the differences in the dark period disappear after SD (Fig. 5). Whereas in Shank3^∆C^ males, the genotype differences present at baseline during the dark period becomes more prominent after SD (Fig. 5). This suggests that SD exacerbates reduction of sleep time for female Shank3^∆C^ in the light period and for male Shank3^∆C^ in the dark period while diminishing the reduction in sleep in the dark period in female Shank3^∆C^ mice. Overall, our study supports baseline and homeostatic sleep deficits in both male and female Shank3^∆C^ mice, with differences being more prominent in female Shank3^∆C^.

Our results are consistent with previous findings from ourselves and others [5, 17, 21, 25]. In addition to confirming that the Shank3^∆C^ mutation produces baseline sleep deficits regardless of sex, we find that it alters the sleep homeostatic response in a sex dependent manner. A reduction of sleep amount or quality (as seen in insomnia) can cause excessive daytime sleepiness leading to an increase in daytime napping during what is typically an active period [14]. Excessive daytime napping or sleepiness has been linked to an increased risk for cardiovascular disease, obesity, and neurological disorders [34, 40, 42] such as Alzheimer’s, Parkinson’s, and ASD [6, 32, 33]. Thus, the decrease in sleep during the rest phase and excessive sleep in the active phase in Shank3^∆C^ females may be indicative of worse sleep disturbances compared to Shank3^∆C^ males who only sleep less during the active phase. Furthermore, while Shank3^∆C^ males show a lack of sleep rebound in response to SD, females display a paradoxical reduction of sleep time in response to SD (Fig. 4). Our findings align with emerging clinic research indicating that sleep disturbances may be a core part of the ASD phenotype but may manifest differently and/or be more severe in autistic females [10, 12].

## Limitations

The present study provides valuable insights however some limitations are present. First, our study is focused on a single mouse model of ASD in adulthood. Shank3^∆C^ mutants were chosen for this study as mice with Shank3 mutations recapitulate multiple features of ASD including deficits in social behavior, cognitive impairments, and key insomnia phenotypes such as sleeping less and delayed seep onset following SD [8, 17, 19, 21, 25, 35]. However, it is possible that other ASD mouse models may display different sleep phenotypes. Second, our study utilized only adult mice. It is possible that the interaction between sex and sleep in ASD is altered in an age-dependent manner. Last, although we find significant main effects of both sex and genotype in both sleep latency and sleep pressure accumulation after SD, we are not sufficiently powered to survive multiple testing in some of our post hoc comparisons, thus limiting our ability to disentangle the effect of sex from genotype for those two measures.

## Perspectives and significance

Females are severely understudied in ASD research, likely because ASD has a diagnosis rate of four males to one female [22]. This male bias has resulted in preclinical and clinical research being skewed towards male specific effects. Identification of sex-specific differences in sleep deficits in this ASD mouse model allows for future research to be aimed at understanding the underlying mechanisms. Mechanistic insight into these sex specific sleep deficits may inform more targeted and effective interventions, improving outcomes for individuals with ASD.

## Supplementary Information

Supplementary Material 1. Additional file 1 includes all supplementary figures (Additional Figure S1-S5) in a word document with captions attached.

Supplementary Material 2. Additional file 2 includes statistical data from the data analysis conducted for the current study in an excel format.

## Data Availability

The datasets generated during the current study are available at https://sleepdata.org/datasets/shank3zz. Code used to analyze the dataset in the current set is available at https://github.com/PeixotoLab/Shank3Z_sexdifferences.
