# Supplementary material for: Sex differences in sleep deficits in mice with an autism-linked Shank3 mutation
Source: Biol Sex Differ. 2024 Oct 28;15:85. doi: 10.1186/s13293-024-00664-6 (PMC11514800; doi:10.1186/s13293-024-00664-6)

**Supplementary Figure S1.** Baseline time in state as percentage of total recording time (TRT) across 24 hours. Wake, NREM sleep and REM sleep during baseline light and dark (black bar) periods. Left panel males, right panel females. WT represented in black, Shank3^ΔC^ mice represented in red (n =7/sex/ genotype). Gray bar represents significant post hoc t-test across genotype within sex with Benjamini-Hochberg correction. Wake: Males dark period p = 0.020, females light period p = 0.010, females dark period p = 0.028. NREM sleep: males dark period p = 0.018, females light period p = 0.009, females dark period p = 0.047. REM sleep: females dark period p = 0.007.

**Supplementary Figure S2.** A-C. Bout number and duration in Wake, NREM sleep, and REM sleep during baseline light period. D-F. Bout number and duration in Wake, NREM sleep, and REM sleep during the dark period. WT represented in black, Shank3^ΔC^ mice represented in red, filled circles represents males, unfilled represents females (n = 7/sex/ genotype). Repeated measure ANOVA, post hoc Benjamini-Hochberg correction, # p-values < 0.05 across sex. * p-values < 0.05 across genotype. 2A bout number males across genotype p = 0.007, females across genotype p = 0.040, Shank3^ΔC^ across sex p = 2.254E-04. 2A bout duration males across genotype p = 0.015, Shank3^ΔC^ across sex p = 0.026. 2B bout number males across genotype p = 0.030. 2B bout duration Shank3^ΔC^ across sex p = 0.008. 2D bout duration females across genotype p = 0.043, Shank3^ΔC^ across sex p = 0.043. 2F bout number males across genotype p = 0.013, females across genotype p = 0.047, Shank3^ΔC^ across sex p = 0.013.

**Supplementary Figure S3.** Recovery day time in state as percentage of total recording time (TRT). Wake, NREM sleep, and REM sleep during baseline light (white) and dark (black) periods, grated area represents the five hours of SD. WT represented in black, Shank3^ΔC^ mice represented in red(n = 7 WT males, 7 WT females, 6 Shank3^ΔC^ males, 7 Shank3^ΔC^ females). Gray bar represents significant post hoc t-test across genotype within sex with Benjamini-Hochberg correction. Wake: males dark period p = 0.002, females light period p = 1.474E-04. NREM sleep: males dark period p = 0.001, females light period p = 1.053E-04

**Supplementary Figure S4.** A-C. Bout number and duration in Wake, NREM sleep, and REM sleep during recovery day light period. D-F. Bout number and duration in Wake, NREM sleep and REM sleep during the dark period. Repeated measure ANOVA, post hoc Benjamini-Hochberg correction, # p-values < 0.05 across sex. * p-values < 0.05 across genotype. WT represented in black, Shank3^ΔC^ mice represented in red, filled represents males, unfilled represents females (n =7 WT males, 7 WT females, 6 Shank3^ΔC^ males, 7 Shank3^ΔC^ females). 4A bout number males across genotype p = 0.022, Shank3^ΔC^ across sex p = 0.026. 4A bout duration males across genotype p = 0.003. 4C bout duration males across genotype p = 0.043, females across genotype p = 0.015. 4E bout number WT across sex p = 0.023. 4E bout number WT across sex p = 0.049.

**Supplementary Figure S5.** Recovery sleep spectral power. Data normalized to total state specific power across 24 hours of baseline for either light period or dark period of recovery sleep day. Light period (ZT 0-12) A-B. Dark period (ZT 13-24) C-D. 95% confidence intervals shaded in red and gray. WT represented in black, Shank3^ΔC^ mice represented in red. top row males (solid lines), bottom row females (dotted lines). (n =7 WT males, 7 WT females, 6 Shank3^ΔC^ males, 7 Shank3^ΔC^ females).


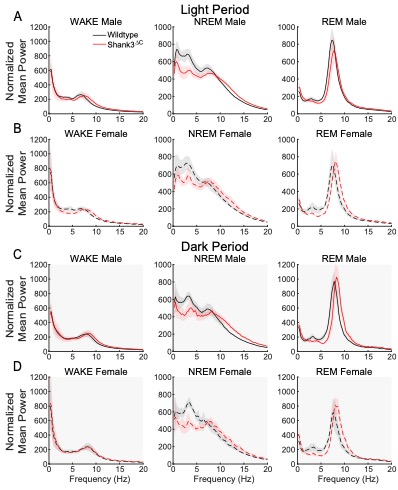

Supplement: Supplementary file 1 — Supplementary Material 1. Additional file 1 includes all supplementary figures (Additional Figure S1-S5) in a word document with captions attached. [file 13293_2024_664_MOESM1_ESM.docx]
